# Avaliação Cardiodinâmica Não Invasiva por Cardiografia de Impedância durante o Teste de Caminhada de Seis Minutos em Pacientes com Insuficiência Cardíaca

**DOI:** 10.36660/abc.20230087

**Published:** 2023-12-14

**Authors:** Leandro Franzoni, Rafael Cechet de Oliveira, Diego Busin, Douglas Jean Preussler Turella, Rochelle Rocha Costa, Marco Aurélio Lumertz Saffi, Anderson Donelli da Silveira, Ricardo Stein

**Affiliations:** 1 Programa de Pós-Graduação em Ciências da Saúde: Cardiologia e Ciências Cardiovasculares UFRGS Porto Alegre RS Brasil Programa de Pós-Graduação em Ciências da Saúde: Cardiologia e Ciências Cardiovasculares ( UFRGS ), Porto Alegre , RS – Brasil; 2 Universidade de Caxias do Sul Caxias do Sul RS Brasil Universidade de Caxias do Sul , Caxias do Sul , RS – Brasil; 3 Universidade de Brasília Programa de Pós-Graduação em Educação Física Porto Alegre RS Brasil Universidade de Brasília - Programa de Pós-Graduação em Educação Física , Porto Alegre , RS – Brasil; 4 Hospital de Clínicas de Porto Alegre Porto Alegre RS Brasil Hospital de Clínicas de Porto Alegre , Porto Alegre , RS – Brasil

**Keywords:** Cardiografia de Impedância, Tolerância ao Exercício, Hemodinâmica

## Abstract

**Fundamento:**

O Teste de Caminhada de seis Minutos (TC6M) é comumente usado para avaliar pacientes com insuficiência cardíaca. No entanto, vários fatores clínicos podem influenciar a distância percorrida pelos pacientes no teste. A cardiografia de impedância (CI) na avaliação morfológica é uma ferramenta útil para avaliar a hemodinâmica cardíaca de maneira não invasiva.

**Objetivo:**

Este estudo teve como objetivo comparar as respostas de aceleração e desaceleração do Débito Cardíaco (DC), da Frequência Cardíaca (FC), e do Volume Sistólico (VS) ao TC6M de indivíduos com insuficiência cardíaca e fração de ejeção reduzida (ICFEr) com as de controles sadios.

**Métodos:**

Este é um estudo transversal observacional. O DC, a FC, o VS e o Índice Cardíaco (IC) foram avaliados antes, durante e após o TC6M por CI. O nível de significância adotado na análise estatística foi 5%.

**Resultados:**

Foram incluídos 27 participantes (13 com ICFEr e 14 controles sadios). A aceleração do DC e da FC foi significativamente diferente entre os grupos (p<0,01 e p=0,039, respectivamente). Encontramos diferenças significativas no VS, no DC e no IC entre os grupos (p<0,01). A regressão linear mostrou uma contribuição deficiente do VS à mudança no DC no grupo com ICFEr (22,9% versus 57,4%).

**Conclusão:**

O principal resultado deste estudo foi o fato de que indivíduos com ICFEr apresentaram valores mais baixos de aceleração do DC e da FC durante o teste de exercício submáximo em comparação a controles sadios. Isso pode indicar um desequilíbrio na resposta autonômica ao exercício nessa condição.

## Graphical abstract

**Figura Central:**
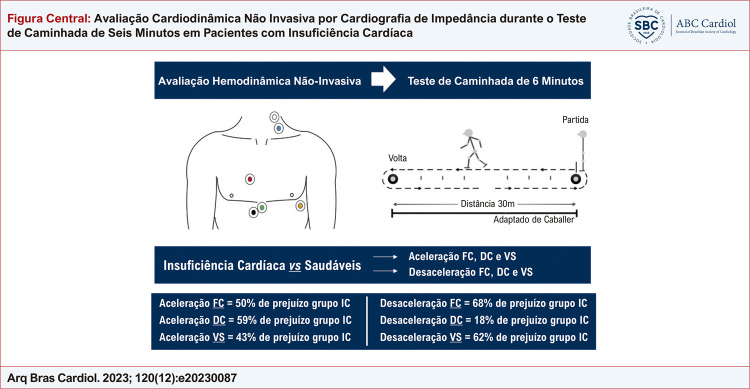
: Avaliação Cardiodinâmica Não Invasiva por Cardiografia de Impedância durante o Teste de Caminhada de Seis Minutos em Pacientes com Insuficiência Cardíaca

## Introdução

A insuficiência cardíaca é uma síndrome complexa que pode ser a última consequência da maioria das doenças cardiovasculares. A redução na contratilidade é uma das principais características da insuficiência cardíaca com fração de ejeção reduzida (ICFEr), em que um débito cardíaco (DC) deficiente resulta em hipoperfusão sistêmica. Essa, combinada com alterações pulmonares, periféricas e neuro-humorais, contribui para uma baixa tolerância à atividade física. ^1 , 2^ Essa capacidade reduzida para a realização de atividades físicas é um dos marcos da doença, comum à maioria dos indivíduos com ICFEr. ^3^

O teste de caminhada de seis minutos (TC6M) é um método amplamente utilizado para avaliar respostas agudas ao exercício autolimitado, em que a distância caminhada constitui um marcador prognóstico comprovado. ^4^ Ttrata-se de um teste simples e de baixo custo, fácil de ser realizado e que não requer treinamento especializado. ^5^ No entanto, estudos mostraram que certas comorbidades, tais como ICFEr, reduzem significativamente a distância percorrida (dependendo do desempenho cardíaco, ou seja da gravidade da doença), requerendo mais avaliações com outras ferramentas para uma melhor informação clínica e prognóstica. ^6^

A cardiografia de impedância (CI) na avaliação morfológica é um método não invasivo que mede, com precisão, o DC, o volume sistólico (VS), a frequência cardíaca (FC), e o índice cardíaco (IC). Pesquisadores clínicos podem usá-la para avaliar tanto indivíduos sadios como aqueles com condições como ICFEr durante o TC6M, obtendo diferentes informações sobre as condições de saúde. ^7 - 10^ Dados da literatura mostram que um aparelho de CI comercialmente disponível, o PhysioFlow® consegue avaliar, com precisão, o pico do DC (em comparação aos métodos de Fick e de termodiluição), tanto em repouso como durante o exercício. ^11 - 13^ Ainda, a CI na avaliação morfológica pode adicionar informação preditiva sobre o pico de consumo de oxigênio (picoVO _2_ ) com uma forte correlação entre picoVO _2_ medido e predito (r = 0,931; p<0,001) por meio dos valores de DC, VS, e de FC obtidos durante o TC6M. ^14^

A CI fornece dados hemodinâmicos úteis durante o TC6M em diferentes condições clínicas. ^15^ No entanto, respostas de aceleração e desaceleração do DC, da FC e do VS ao TC6M ainda precisam ser demonstradas em indivíduos com ICFEr; essas variáveis podem representar desequilíbrios autonômicos no esforço e na recuperação. ^7 , 11^ Assim, este estudo teve como objetivo avaliar as respostas de aceleração e desaceleração do DC, da FC e do VS ao TC6M em pacientes com ICFEr e em controles sadios. Nosso objetivo secundário foi avaliar o comportamento hemodinâmico (pelo DC, FC, VS e IC) antes, durante e após o TC6M, que ainda não foi descrito na literatura.

## Métodos

### Delineamento experimental

Este é um estudo transversal observacional com dois grupos, um composto de indivíduos sadios (grupo controle - GC), e o outro de pacientes com ICFEr (número de aprovação pelo comitê de ética institucional 180651). Nossa amostra foi selecionada por conveniência.

Os critérios de inclusão foram definidos como sinais e sintomas clínicos de IC, avaliados por ecocardiografia, e uma fração de ejeção <40% em pacientes em tratamento farmacológico padrão adequado. Os pacientes incluídos estavam estáveis por pelo menos três meses (sem internação hospitalar, atendimentos de emergência por ICFEr descompensada ou mudança na terapia medicamentosa) e eram seguidos regularmente em uma clínica especializada em IC. Os pacientes com doenças pulmonares e doenças vasculares foram excluídos da nossa amostra. Os participantes do grupo controle não apresentavam nenhuma doença cardíaca e estavam sedentários por pelo menos seis meses. Os controles incluídos foram pareados por idade com os participantes com ICFEr. Os pacientes foram convidados a participar do estudo, e os procedimentos foram explicados a eles. ^15^

Os participantes do grupo IC foram rastreados e recrutados por busca ativa nos prontuários médicos do ambulatório de IC do hospital. O grupo controle foi composto de trabalhadores do hospital, e o contato foi feito por telefone usando uma lista pré-estabelecida. Todos os participantes que foram convidados a participar no estudo leram e assinaram um termo de consentimento antes da coleta de dados.

### Cardiografia de Impedância na avaliação morfológica

A Impedância (Z) é uma medida da resistência a uma corrente elétrica. A CI é um método de medida para avaliar o fluido do tórax. A partir da determinação da corrente e da voltagem, mudanças na impedância resultam em mudanças no volume sanguíneo que passa pelo tórax. ^16^ A variação na voltagem (Z) é filtrada pelo programa do aparelho de CI para evitar a influência de variações no volume inspirado e expirado, e nos fluidos do tórax, ou outros fatores (tais como obesidade ou posição do eletrodo), que afetam a CI convencional. ^11^ Estudos anteriores mostraram fortes correlações entre a medida de CI e métodos invasivos de avaliação hemodinâmica ^17^ tanto em repouso como durante o exercício. ^11 , 12^ A CI pode ser empregada como uma ferramenta diagnóstica, ^18^ e tem sido usada como um preditor de prognóstico cardiovascular. ^19^

**Figura 1:**
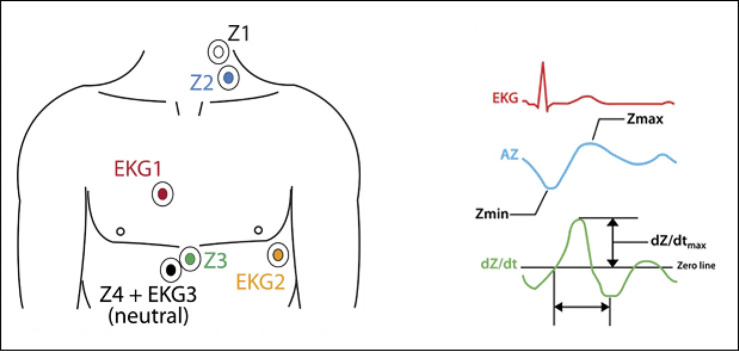
– Esquema ilustrando o posicionamento dos eletrodos do PhysioFlow ® e registros correspondentes.

O peso e a altura de cada participante foram medidos. A pele de cada participante foi preparada (depilada com uma lâmina de barear descartável e gel abrasivo, higienizada com álcool, e seca) para o posicionamento do eletrodo. No total, seis eletrodos (nunca usados anteriormente) de monitoramento cardíaco (FS-50 Skintact, Skintact®, Áustria) foram conectados por fios a um aparelho portátil de CI (PhysioFlow® PF07 Enduro™, Paris, França; 11.5 x 8.5 x 1.8 cm; peso 200g), o qual estava conectado a um adaptador Bluetooth. Os eletrodos foram colocados na região lateral esquerda do pescoço dos participantes, no centro do esterno, em posições padrões V1 e V6, e paralelamente à coluna vertebral, na altura do processo xifoide ( Figura 1 ). O aparelho e os fios foram estabilizados com uma fita de nylon na cintura do paciente para se reduzir o ruído. O sistema foi calibrado com base em 30 batimentos consecutivos medidos em repouso, estabelecendo, assim, a morfologia basal do paciente e os valores hemodinâmicos de repouso.

### Teste de Caminhada de Seis Minutos

O TC6M foi realizado de acordo com as diretrizes da *American Thoracic Society* . ^20^ O teste foi conduzido em um corredor de 30 metros. Os participantes foram orientados a andarem a maior distância possível no intervalo de tempo de seis minutos. A distância percorrida foi registrada e expressa em metros.

### Medidas hemodinâmicas

Os dados da CI foram gravados continuamente a cada batimento. Valores falsos foram manualmente excluídos. O DC foi medido em litros por minuto (L·min ^-1^ ); o VS em mililitros (mL); a FC em batimentos por minuto (bpm), e o IC em litros por minuto por área da superfície corporal em metros ao quadrado (L·min ^-1^ ·m ^-2^ ).

Para a análise, os valores basais (definidos como a média das medidas obtidas nos dois minutos que precederam a avaliação, com os pacientes de pé, por questões práticas), valores máximos obtidos durante o TC6M, deltas (diferença entre o valor máximo e o valor basal), e os valores no primeiro minuto de recuperação foram usados.

### Aceleração e desaceleração do DC, FC e VS

A aceleração foi definida como a diferença entre os valores de repouso e a média de todos os valores obtidos durante o primeiro minuto do TC6M, enquanto a desaceleração foi definida como a diferença entre os valores medidos no final do teste e a média de todos os valores obtidos durante o primeiro minuto do TC6M. Essas variáveis foram coletadas durante o primeiro minuto de caminhada (aceleração) e o primeiro minuto de recuperação (desaceleração), uma vez que esses são os momentos do TC6M em que as mudanças hemodinâmicas mais acentuadas ocorrem. A variabilidade das respostas hemodinâmicas ao primeiro minuto de exercício é representada pela aceleração, isto é, demanda cardiovascular aumentada. A variabilidade da resposta imediatamente após o exercício e durante o início da recuperação é representada pela desaceleração. Todos os participantes foram monitorados por 18 minutos – seis minutos de pé, seis minutos de teste de caminhada, e seis minutos durante a recuperação.

### Análise estatística

Os dados foram apresentados em média e desvio padrão (DP) ou mediana e intervalo interquartil (IIQ), de acordo com o teste de normalidade. As variáveis categóricas foram apresentadas como frequência (absoluta e relativa) e o teste do qui-quadrado foi usado para avaliar as diferenças entre os grupos quanto a essas variáveis. A distribuição dos dados foi avaliada pelo teste de Shapiro-Wilk. Para as comparações entre grupos, o teste t independente ou o teste de Mann-Whitney foi usado conforme apropriado. A correlação de Pearson foi aplicada para avaliar a força da associação entre as variáveis. Regressão linear multivariada, com as mudanças no DC como variável dependente, foi usada para identificar a contribuição das mudanças na FC e no VS. Todas as cinco premissas necessárias para o uso da análise de regressão linear foram verificadas (relação linear, normalidade multivariada, pouca ou nenhuma multicolinearidade, ausência de autocorrelação, homoscedasticidade).

O alfa foi definido como <0,05 para indicar significância estatística. As análises estatísticas foram realizadas no programa SPSS, versão 20.0 (IBM; ARMONK, NY, EUA).

## Resultados

### Caracterização da amostra

**Figura 2:**
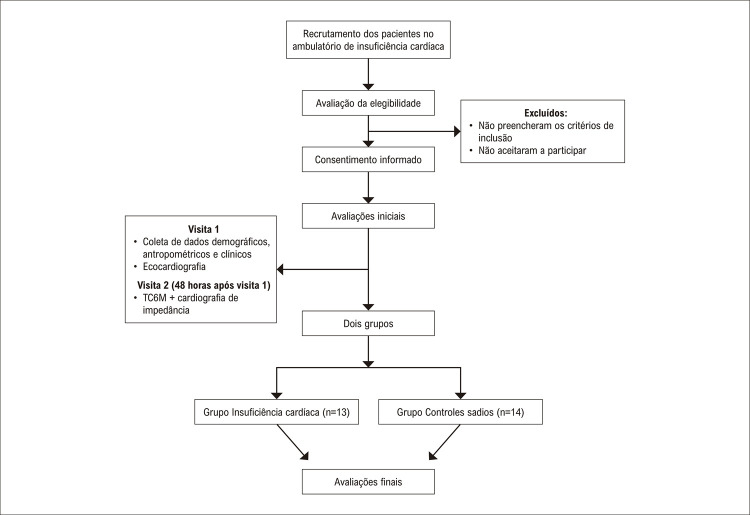
– Fluxograma do estudo.

O fluxograma de rastreamento, eligibilidade, e avaliação dos pacientes está ilustrado na Figura 2 . A Tabela 1 apresenta as características basais dos participantes.

**Tabela 1:** – Características basais do grupo de pacientes com Insuficiência Cardíaca e Fração de Ejeção reduzida (ICFEr) e do Grupo Controle (GC)

Características	GC = 14	ICFEr = 13	p
Idade (anos)	65 ± 5	64 ± 8	0,66
Altura (cm)	167 ± 11	171 ± 12	0,28
Peso (kg)	72 ± 15	79,5 ± 9	0,45
Fação de ejeção (%)	63,4 ± 6,23	32,7 ± 5,25	0,17
Mulheres	0 (0)	2 (15,4)	0,78
**Etnia, n (%)**			
Negra	3 (21,4)	3 (23)	1,00
Branca	11 (78,6)	10 (77)	0,87
**Classe NYHA, n (%)**			
I	-	1 (7,7)	-
II	-	9 (69,2)	-
III	-	3 (23)	-
**Etiologia da IC, n (%)**			
Isquêmica	-	4 (30,8)	-
Não-isquêmica	-	9 (69,2)	-
Fração de ejeção do ventrículo esquerdo (%)	-	32,7 ± 5,2	-
**Medicamentos, n (%)**			
Varfarina	-	1 (7,7)	-
Inibidores de ECA/BRAs	-	13 (100)	-
Betabloqueadores	-	13 (100)	-
Diuréticos	-	7 (53,7)	-
Digoxina	-	7 (53,8)	-
Isossorbida	-	2 (15,4)	-
Antidiabéticos	-	4 (30,7)	-
Aspirina	-	3 (23)	-
Sinvastatina	-	4 (30,7)	-

NYHA: New York Heart Association; ECA: enzima conversora de angiotensina; BRAs: bloqueadores do receptor da angiotensina; dados são apresentados em média e desvio padrão ou frequência (absoluta e relativa).

### Teste de caminhada de seis minutos

#### Aceleração e desaceleração – DC, FC e VS

**Figura 3:**
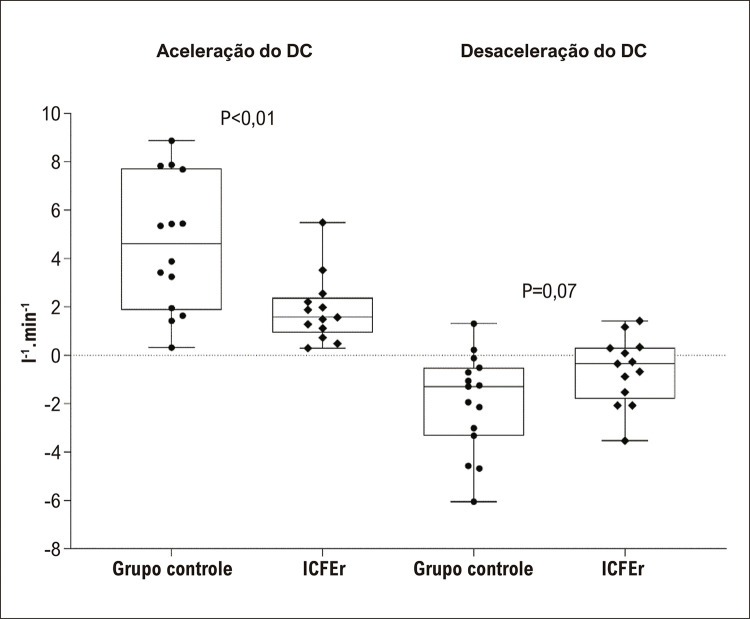
– Comparação das respostas de aceleração e desaceleração do Débito Cardíaco (DC) ao teste de caminhada de seis minutos entre pacientes com Insuficiência Cardíaca e Fração de Ejeção reduzida (ICFEr) e indivíduos sadios (grupo controle).

**Figura 4:**
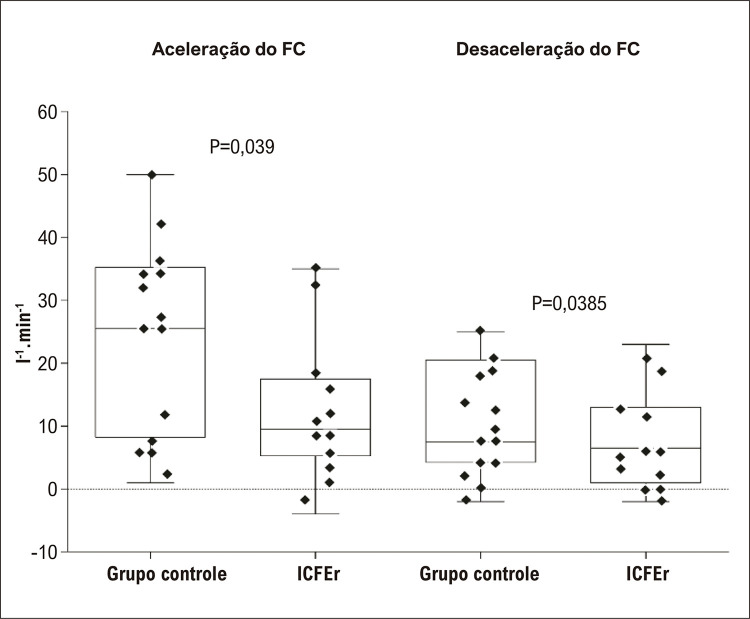
– Comparação das respostas de aceleração e desaceleração da Frequência Cardíaca (FC) ao teste de caminhada de seis minutos entre pacientes com Insuficiência Cardíaca e Fração de Ejeção reduzida (ICFEr) e indivíduos sadios (grupo controle).

A aceleração do DC foi significativamente diferente entre os grupos (ICFEr 1,89 ± 1,39 l.min ^-1^ .s ^-1^ ; GC: 4,59 ± 2,75 l.min ^-1^ .s ^-1^ , p<0,01). Em contraste, a desaceleração do DC não foi diferente entre os grupos (ICFEr: 0,62 ± 1,39 l.min ^-1^ .s ^-1^ ; GC: 1,94 ± 2,11 l.min ^-1^ .s ^-1^ , p=0,07). (Figure 3). Ainda, a aceleração da FC foi significativamente diferente entre os grupos (ICFEr: 12 ± 12 bpm.s ^-1^ ; GC: 24 ± 15 bpm.s ^-1^ , p=0,039), e a desaceleração da FC não foi diferente entre os grupos (9 ± 8 bpm.s ^-1^ ; GC 11 ± 9 bpm.s ^-1^ , p=0,385) ( Figura 4 ). Em contraste, tanto a aceleração como a desaceleração do VS não foram diferentes entre os grupos (ICFEr: 15,51 ± 14,38 ml.s ^-1^ ; GC: 25,12 ± 15,65 ml.s ^-1^ , p=0,110 e ICFEr 3,29 ± 9,01 ml.s ^-1^ ; GC: 8,85 ± 16,98 ml.s ^-1^ , p=0,304).

#### Medidas convencionais

A Tabela 2 apresenta os desfechos tradicionalmente medidos durante o TC6M. Em comparação aos participantes do GC, os pacientes com ICEFr caminharam uma distância mais curta, com uma FC similar em repouso e durante o teste, mas mostraram uma FC mais baixa durante o primeiro minuto de recuperação. Os pacientes com ICFEr apresentaram picos de VS e IC significativamente mais baixos ( Tabela 3 ).

**Tabela 2:** – Parâmetros tradicionais medidos durante o teste de caminhada de seis minutos

	GC (n= 14)	ICFEr = 13	p

	Média ± DP ou Mediana (IIQ)	Média ± DP ou Mediana (IIQ)	
Distância percorrida (m)	559,50 ± 61,36	395,50 ± 87,63	< 0,01
FC basal (bpm)	72 ± 8	71 ±9	0,927
FC máxima (bpm)	112 (108;138)	116 (91;146)	0,607
Mudança na FC (bpm)	43 (31;67)	48 (23;65)	0,837
Recuperação na FC em 1 minuto (bpm)	15 ± 9	8 ± 11	0,097

Bpm: batimentos por minuto; FC: frequência cardíaca; GC: grupo controle; ICFEr: insuficiência cardíaca com fração de ejeção reduzida; IIQ: intervalo interquartil.

**Tabela 3:** – Parâmetros hemodinâmicos medidos por cardiografia de impedância durante o teste de caminhada de seis minutos (TC6M)

	GC (n= 14)	ICFEr = 13	p

	Média ± DP ou Mediana (IIQ)	Média ± DP ou Mediana (IIQ)	
VS basal (mL)	83,77 ± 16,48	51,28 ± 13,78	< 0,01
Máximo VS (mL)	147,03 ± 28,16	100,13 ± 27,96	< 0,01
Mudança no VS (mL)	60,36 (47,41; 69,82)	41,76 (30,76; 67,90)	0,13
Recuperação do VS em 1 min (mL)	18,49 ± 13,38	5,34 ± 7,55	< 0,01
DC basal (l.min ^-1^ )	5,94 ±1,05	3,64 ± 1,03	< 0,01
DC Máximo (l.min ^-1^ )	16,35 (15,20; 17,70)	10,30 (7,31; 14,83)	< 0,01
Mudança do DC (l.min ^-1^ )	10,15 (8,82; 12,20)	7,10 (3,64; 10,90)	0,05
IC basal (l.min ^-1^ .m ^-2^ )	2,97 (2,70; 3,41)	1,99 (1,68; 2,19)	< 0,01
IC máximo (l.min ^-1^ .m ^-2^ )	8,67 ± 2,46	6,12 ± 1,90	< 0,01
Mudança no IC (l.min ^-1^ .m ^-2^ )	5,61 ± 2,54	4,09 ± 2,03	0,09
Recuperação do IC em 1 min (l.min ^-1^ .m ^-2^ )	1,77 ± 0,92	0,58 ± 0,70	< 0,01

IC: índice cardíaco; VS: volume sistólico; DC: débito cardíaco, GC: grupo controle; ICFEr: insuficiência cardíaca com fração de ejeção reduzida; IIQ: intervalo interquartil; min: minuto.

#### Distância percorrida no TC6M, variáveis hemodinâmicas e classe funcional

Quanto maior foi a aceleração do DC, maior foi a distância percorrida durante o TC6M (r=0,49, p=0,01). IC basal (r=0,60, p<0,01), pico do IC (r=0,67, p<0,01), ΔCI (r=0,63, p<0,01), IC durante o primeiro minuto de recuperação (r=0,68, p<0,01), bem como o VS durante o primeiro minuto de recuperação (r=0,50, p<0,01) foram significativamente correlacionados com a distância percorrida durante o TC6M.

A distância percorrida durante o TC6M correlacionou-se com a FC no primeiro minuto de recuperação (r=0,68, p<0,01) e classe NYHA (r=0,62, p<0,01). Além disso, o VS basal e o pico do VS correlacionaram-se significativamente com a distância percorrida durante o teste (r=0,51, p=0,01, r=0,60, p<0,01, respectivamente). O DC basal, o pico do DC e ΔCO também se correlacionaram significativamente com a distância percorrida no TC6M (r=0,52, p<0,01, r=0,67, p<0,01, r=0,61, p<0,01, respectivamente).

## Contribuição das variáveis para a alteração no débito cardíaco

Mudanças na FC explicaram 64,3% da ΔCO no grupo controle e 70,3% nos pacientes com ICFEr. A contribuição da ΔVS foi de 57.4% no grupo controle e somente 22,9% no grupo com ICFEr. De acordo com coeficientes de regressão β, para cada unidade de FC alterada, houve uma mudança na ΔCO de 1,121 unidades no grupo controle e de 0,92 unidade no grupo ICFEr. Em relação à ΔVS, cada unidade de FC alterada, houve uma mudança na ΔSV de 1,162 unidades no grupo controle e de 0,91 unidade no grupo ICFEr.

## Discussão

No presente estudo, comparamos respostas cardiodinâmicas ao TC6M entre pacientes com ICFEr e indivíduos sadios. Nosso principal achado é que os pacientes com ICFEr mostraram respostas hemodinâmicas diminuídas ao TC6M, principalmente a aceleração do DC e da FC (em comparação aos controles). Além disso, encontramos diferenças significativas na distância caminhada durante o TC6M entre os grupos, reforçando a esperada redução na capacidade funcional dos indivíduos com ICFEr. Ainda, durante a recuperação, a resposta da FC foi mais baixa no grupo de pacientes com ICFEr, sugerindo uma deficiência no sistema nervoso autônomo parassimpático que realmente ocorre nesses pacientes ( Figura Central ).

Um desequilíbrio autonômico, caracterizado pela predominância simpática, é uma característica clássica da ICFEr, com consequências clinicamente relevantes. Essas incluem progressão da doença, desenvolvimento ou deterioração da intolerância ao exercício, remodelamento ventricular e arritmias, e morte prematura. Os mecanismos subjacentes desses processos e sua ocorrência ao longo do tempo ainda necessitam ser descritos.

Em nosso conhecimento, vários estudos avaliaram o perfil hemodinâmico de diferentes doenças durante o TC6M. ^15 , 21 - 23^ Um estudo ^22^ avaliou a aceleração e a desaceleração do DC (mas não do VS) na hipertensão pulmonar. Essas variáveis são importantes para a ICFEr, uma vez que elas representam um desequilíbrio nas respostas autonômicas ao esforço e à recuperação. ^24 , 25^ Encontramos uma diferença significativa na aceleração do DC entre os grupos (p<0,01) mas não na sua desaceleração (p=0,07), e um comportamento similar na aceleração (p=0,039) e na desaceleração (p=0,385). A aceleração e a desaceleração tanto do DB como da FC foram mais baixas no grupo com ICFEr em comparação aos controles; esses pacientes apresentam um déficit cronotrópico devido à doença em si, e ao efeito farmacológico dos betabloqueadores (todos os pacientes estavam recebendo tratamento com betabloqueadores).

Uma vez que a aceleração e a desaceleração podem representar uma ativação simpática e parassimpática, respectivamente, a ICFEr apresenta uma maior ativação simpática e um desequilíbrio simpatovagal, o que corrobora os achados de nosso estudo, mostrando uma aceleração tanto do DC como da FC no grupo de pacientes com ICFEr. ^26 , 27^ Modelos animais mostraram que a estimulação simpática e um reflexo cardiovascular anormal contribuem para ativar o sistema nervoso simpático na ICFEr. ^28^ Por outro lado, pouco se sabe sobre o papel da atividade nervosa parassimpática nessa condição. Hu et al. ^26^ mostraram que a desaceleração da FC é um preditor independente de infarto agudo do miocárdio e morte súbita na ICFEr, constituindo um preditor mais forte que a fração de ejeção ventricular esquerda e medidas convencionais da variabilidade da FC. Ainda, os mesmos autores ^26^ avaliaram somente as respostas de aceleração e desaceleração da FC. Assim, nosso estudo é o primeiro a avaliar a aceleração e a desaceleração do DC, da FC, e do VS na ICFEr. Apesar de não termos conseguido demonstrar uma diferença significativa na desaceleração do DC entre os grupos, encontramos uma nítida tendência (p=0,07) de perda nas respostas no grupo ICFEr.

Quanto à regulação da FC, uma disfunção ventricular na ICFEr pode desencadear mecanismos compensatórios distintos, que inicialmente aumenta a ativação neuro-hormonal do sistema nervoso simpático e do sistema renina-angiotensina-aldosterona. ^29^ Contudo, uma exposição prolongada à ativação simpática pode inibir a atividade de receptores beta-adrenérgicos, contribuindo para respostas inotrópicas, que podem prejudicar a recuperação da FC após o exercício. ^30^ Esses dados são corroborados pelos nossos resultados. ^2^

Encontramos valores de VS basal e de pico mais baixos nos pacientes com ICFEr em comparação aos controles. Tanto o comportamento em repouso como no exercício no grupo com ICFEr representam uma perda na contração ventricular, isto é, uma redução no VS em cada sístole. ^31^ Nos indivíduos sadios, o princípio de Frank-Starling descreve um mecanismo fisiológico que aumenta o VS para compensar a redução inicial da contração ventricular. ^32^ Por outro lado, os indivíduos com ICFEr apresentam falhas nesse mecanismo. A consequente redução na reserva cardiovascular prejudica e reduz a contratilidade ventricular, diminuindo o VS. ^33^ Esse fenômeno também corrobora os achados de nosso estudo.

Ainda, observamos diferenças significativas nos valores basais e de pico do IC e do DC entre os grupos, que pode ser explicado por mecanismos compensatórios da FC e do VS. ^21^ Encontramos que a disfunção ventricular reduziu a IC e o DC em pacientes com ICFEr, levando a mecanismos que inicialmente aumentam essas variáveis para manter a perfusão no órgão-alvo. ^34^ Contudo, após uma longa exposição a eles, o miocárdio sofre remodelamento, reduzindo a capacidade inotrópica ventricular e o VS, consequentemente afetando tanto o IC como o DC. ^2^

Quando submetidos ao exercício submáximo, indivíduos com ICFEr mostram respostas diminuídas tanto para o aumento como para a diminuição no VO2 em indivíduos sadios. ^35^ Esses pacientes também podem apresentar hipertensão pulmonar leve, o que pode explicar o comportamento do DC e do VS. ^22 , 36^ O comportamento do DC pode então ser devido ao desequilíbrio nas respostas autonômicas, afetando o VS. ^37^

Nós testamos se os parâmetros hemodinâmicos, avaliados pela CI, tinham correlação com a distância percorrida durante o TC6M, e encontramos que quanto maior a aceleração do DC, maior a distância percorrida (r=0,49; p=0,01). Ainda, os indivíduos com a recuperação mais rápida na FC após o teste foram aqueles que caminharam distâncias mais longas. Embora esse não tenha sido o principal objetivo do estudo, encontramos que a avaliação hemodinâmica pela CI durante o TC6M pode fornecer resultados interessantes que refletem diretamente a capacidade funcional. Além da distância no TC6M, encontramos que a classe funcional NYHA teve uma boa associação com o DC máximo, a desaceleração do DC, e a FC durante o primeiro minuto de recuperação após o teste. ^38^ Conforme o esperado, encontramos diferenças nas distâncias percorridas no TC6M entre os grupos (p<0,01), corroborando um estudo prévio. ^39^

Finalmente, a regressão linear mostrou uma contribuição deficiente do VS (22,9%) às mudanças no DC em pacientes com ICFEr, e valores normais nos controles sadios (57,4% de contribuição do VS à mudança no DC). De fato, o pulso de oxigênio, um indicador do VS, foi mais baixo nos pacientes com ICFEr (em comparação aos controles sadios), conforme descrito em estudos prévios, A disfunção no VS, como representado pelo baixo pulso de oxigênio, pode refletir uma distribuição sistêmica insuficiente de oxigênio durante o exercício e/ou uma utilização deficiente de oxigênio devido à uma redução na função mitocondrial. ^40 , 41^

O estudo teve algumas limitações. Primeiro, o tamanho pequeno da amostra pode ter limitado a capacidade de detectarmos diferenças significativas. No entanto, o principal objetivo deste estudo fisiológico foi avaliar o comportamento hemodinâmico de indivíduos com ICFEr durante o TC6M, compará-lo com o de indivíduos sadios, e determinar a contribuição relativa das variações do DC, do VS, e da FC durante as fases do teste de caminhada. Segundo, nosso estudo não foi delineado para realizar testes de correlação ou associação dos parâmetros de CI com a distância percorrida, classe funcional NYHA ou fração de ejeção.

Embora o TC6M seja seguro, de baixo custo, e facilmente usado para avaliar a capacidade funcional em pacientes com ICFEr, ^20 , 39^ (por exemplo, o teste não fornece dados diretos do comportamento hemodinâmico). Assim, novas tecnologias seriam importantes para adicionar informações aos achados do TC6M e ajudar no tratamento dessa terrível síndrome. Ainda, avanços tecnológicos permitiram o desenvolvimento de um aparelho portátil para medir, de maneira não invasiva e em tempo real, uma ampla gama de parâmetros hemodinâmicos, tais como DC, VS, FC e IC.

## Conclusão

Este é o primeiro estudo que demonstra as respostas hemodinâmicas de aceleração e desaceleração do DC, FC, e VS por meio da CI em pacientes com ICFEr durante o TC6M. Indivíduos com ICFEr apresentaram aceleração do DC e da FC deficiente durante exercícios submáximos em comparação a controles sadios, o que pode representar um desequilíbrio na resposta autonômica ao esforço. Mais estudos são necessários para testar se mudanças no DC, na FC, no VS e no IC durante o TC6M podem fornecer informações sobre o prognóstico da doença.
