# Supplementary material for: Avaliação Cardiodinâmica Não Invasiva por Cardiografia de Impedância durante o Teste de Caminhada de Seis Minutos em Pacientes com Insuficiência Cardíaca
Source: Arq Bras Cardiol. 2023 Dec 14;120(12):e20230087. [Article in Portuguese] doi: 10.36660/abc.20230087 (PMC10789369; doi:10.36660/abc.20230087)
Supplement: Translation

# Translation: Non-Invasive Assessment of Cardiodynamics by Impedance Cardiography during the Six-Minute Walk Test in Patients with Heart Failure

**Authors:** Leandro Franzoni, Rafael Cechet de Oliveira, Diego Busin, Douglas Jean Preussler Turella, Rochelle Rocha Costa, Marco Aurélio Lumertz Saffi, Anderson Donelli da Silveira, Ricardo Stein

**Affiliations:** 1 Brasil Programa de Pós-Graduação em Ciências da Saúde: Cardiologia e Ciências Cardiovasculares (UFRGS), Porto Alegre, RS – Brasil; 2 Brasil Universidade de Caxias do Sul, Caxias do Sul, RS – Brasil; 3 Brasil Universidade de Brasília - Programa de Pós-Graduação em Educação Física, Porto Alegre, RS – Brasil; 4 Brasil Hospital de Clínicas de Porto Alegre, Porto Alegre, RS – Brasil

**Keywords:** Impedance Cardiography, Exercise Tolerance, Hemodynamics

## Abstract

**Background:**

The six-minute walk test (6MWT) is commonly used to evaluate heart failure (HF) patients. However, several clinical factors can influence the distance walked in the test. Signal-morphology impedance cardiography (SM-ICG) is a useful tool to noninvasively assess hemodynamics.

**Objective:**

This study aimed to compare cardiac output (CO), heart rate (HR), and stroke volume (SV) acceleration and deceleration responses to 6MWT in individuals with HF and reduced ejection fraction (HFrEF) and healthy controls.

**Methods:**

This is a cross-sectional observational study. CO, HR, SV and cardiac index (CI) were evaluated before, during, and after the 6MWT assessed by SM-ICG. The level of significance adopted in the statistical analysis was 5%.

**Results:**

Twenty-seven participants were included (13 HFrEF and 14 healthy controls). CO and HR acceleration significantly differed between groups (p<0.01; p=0.039, respectively). We found significant differences in SV, CO and CI between groups (p<0.01). Linear regression showed an impaired SV contribution to CO change in HFrEF group (22.9% versus 57.4%).

**Conclusion:**

The main finding of the study was that individuals with HFrEF showed lower CO and HR acceleration values during the submaximal exercise test compared to healthy controls. This may indicate an imbalance in the autonomic response to exercise in this condition.

## Introduction

Heart failure (HF) is a complex syndrome which may be the ultimate consequence of most cardiovascular diseases. Contractility reduction is one of the core features of HF with reduced ejection fraction (HFrEF), in which cardiac output (CO) impairment results in systemic hypoperfusion. This, combined with pulmonary, peripheral, and neurohumoral changes, contributes to a low tolerance to physical activity. ^1 , 2^ This reduced capacity to perform physical activities is one of the hallmarks of this disease, common to most individuals with HFrEF. ^3^

The six-minute walk test (6MWT) is a widely used method to evaluate acute responses to self-limited exercise, in which the walked distance constitutes a proven prognostic marker. ^4^ Furthermore, it is a simple and inexpensive test, easy to perform and does not require specialized training. ^5^ However, studies have shown that certain comorbidities, such as HFrEF, significantly reduce the distance walked (depending on cardiac performance, *i.e* ., disease severity), requiring further assessment using other tools for better clinical and prognostic information. ^6^

Signal-morphology impedance cardiography (SM-ICG) is a noninvasive method which accurately measures CO, stroke volume (SV), heart rate (HR), and cardiac index (CI). Clinical researchers can use it to assess both healthy individuals and those with conditions such as HFrEF during 6MWT, adding different information about health conditions. ^7 - 10^ The literature has shown that a commercially available SM-ICG device (PhysioFlow ^®^ ) can accurately assess peak CO (when compared to Fick’s and thermodilution methods) both at rest and during exercise. ^11 - 13^ Furthermore, SM-ICG can add predictive information on peak oxygen consumption (VO _2_ peak) with a strong correlation between measured and predicted VO _2_ peak (r = 0.931, p<0.001) by values obtained during 6MWT for CO, SV, and HR. ^14^

SM-ICG provides useful hemodynamic data during 6MWT in different clinical conditions. ^15^ However, CO, HR, and SV acceleration and deceleration responses to 6MWT are yet to be shown in HFrEF individuals; these variables may represent autonomic imbalances in exertion and recovery. ^7 , 11^ Therefore, this study primarily aimed to evaluate CO, HR, and SV acceleration and deceleration responses to 6MWT in patients with HFrEF and in healthy controls. Our secondary objective was to assess hemodynamic behavior (through CO, HR, SV, and CI) before, during, and after 6MWT, which is still unavailable in the literature.

## Methods

### Experimental design

This is a cross-sectional observational study of two groups, one composed of healthy individuals (control group - CG), and the other, of HFrEF patients (institutional review boards number 180651). Our sample was selected by convenience.

Inclusion criteria were defined as HF clinical signs and symptoms, assessed by echocardiography, and an ejection fraction <40% while on optimal pharmacological treatment. The patients included were stable for at least three months (no hospitalization, emergency visits due to decompensated HFrEF or change in drug therapy) and regularly followed at a specialized HF clinic. Patients with lung diseases and peripheral vasculopathy were excluded from our sample. Participants in the control group had no heart disease of any type and were sedentary for at least six months. The enrolled controls were matched for age with participants with HFrEF. Patients were invited to participate in this study, and the study procedures were explained to them. ^15^

Participants in the HF group were screened and recruited through an active search in the medical records of the HF outpatient clinic at the hospital. The control group consisted of individuals working at the hospital, and contact was made via telephone using a pre-established list. All participants who were invited to participate in the study, read and signed a written consent form before data collection.

### Signal-Morphology Impedance Cardiography

Impedance (Z) is a measure of resistance to an electric current. SM-ICG is a measurement method to evaluate thoracic fluid content. From the determination of current and voltage, changes in impedance result in changes in blood volume passing through the thorax. ^16^ Impedance variation (ΔZ) is filtered by the SM-ICG device software to avoid the influence of variations in inspired and expired volume, and chest fluids, or other factors (such as obesity or electrode position), which impairs conventional ICG. ^11^ Previous studies have shown strong correlations between SM-ICG measurement and invasive hemodynamic assessments ^17^ both at rest and during exercise. ^11 , 12^ SM-ICG can be applied as a diagnostic tool, ^18^ and it has been used as a predictor of cardiovascular prognosis. ^19^

Each participant’s height and weight were measured. Their skin was prepared (shaved with a disposable razor blade and abrasive gel, sanitized with alcohol, and dried) for electrode placement. In total, six unused electrodes for cardiac monitoring (FS-50 Skintact, Skintact ^®^ , Austria) were attached by wires to a portable SM-ICG device (PhysioFlow ^®^ PF07 Enduro™, Paris, France; 11.5 x 8.5 x 1.8 cm; weight 200g), which was connected to a Bluetooth adapter. The electrodes were placed on the left side of participants’ neck, in the supraclavicular fossa, in the middle of the sternum, at standard ECG positions V1 and V6, and parallel to the spine at the height of the xiphoid process ( Figure 1 ). Wires and the device were stabilized with a nylon strap at patients’ waist to reduce noise. The system was calibrated based on 30 consecutive heartbeats measured at rest, thereby establishing patients’ baseline morphology and resting hemodynamic values.

**Figure 1:** – Schematical PhysioFlow ® electrode positions and corresponding recordings.

### Six-minute walk test

The 6MWT was performed in accordance with the American Thoracic Society guidelines. ^20^ The test was conducted on a 30-meter corridor. Participants were asked to walk as long as possible in a six-minute timeframe. Walked distance was recorded and expressed in meters.

### Hemodynamic measurements

SM-ICG data were continuously recorded beat by beat. False values were manually excluded. CO was measured in liters per minute (L·min ^-1^ ); SV, in milliliters (mL); HR, in beats per minute (bpm); and CI, in liters per minute per square meter of body surface area (L·min ^-1^ ·m ^-2^ ).

For analysis, baseline values (defined as the mean of measures obtained in the two minutes before the evaluation, with patients standing, for practical reasons), maximum values obtained during 6MWT, deltas (difference between maximum and baseline values), and values within the first minute of recovery were used.

#### CO, HR, and SV acceleration and deceleration

Acceleration was defined as the difference between resting values and the mean of all values obtained during the first minute of 6MWT, whereas deceleration was defined as the difference between the measurements at the end of the test and the mean of all values obtained during the first minute of recovery. These variables were collected during the first minute of walking (acceleration) and the first minute of recovery (deceleration) since these are the moments of the 6MWT in which the most pronounced hemodynamic changes occur. The variability of hemodynamic responses to the first minute of exercise is represented by acceleration, *i.e* ., increased cardiovascular demand. The response variability immediately after the exercise ends and during recovery onset is represented by deceleration. All participants were monitored for 18 minutes (six minutes standing, six minutes of the walking test, and six minutes of standing recovery).

## Statistical analysis

Data were shown in mean and standard deviation (SD) or median and interquartile range (IQR), according to the normality test. Categorical variables were presented as frequency (absolute and relative) and the chi square test was used to assess the differences between groups for these variables. Data distribution was assessed by the Shapiro-Wilk test. For between-group comparisons, the independent *t* -test or the Mann-Whitney U test was used as appropriate. Pearson correlation was applied to assess association strength between variables. Multivariate linear regression, with CO changes as the dependent variable, was used to identify the contribution of HR and SV changes. All the five assumptions necessary for the use of linear regression analysis were verified (linear relationship; multivariate normality; no or little multicollinearity; no autocorrelation; homoscedasticity).

Alpha was defined as <0.05 to indicate statistical significance. Statistical analyses were performed in SPSS, version 20.0 (IBM; ARMONK, NY, USA).

## Results

### Sample characterization

The flowchart of patient screening, eligibility, and assessment is shown in Figure 2 . Table 1 shows participants’ baseline characteristics.

**Figure 2:** – Study flowchart

**Tabela 1:** – Características basais do grupo de pacientes com Insuficiência Cardíaca e Fração de Ejeção reduzida (ICFEr) e do Grupo Controle (GC)

Characteristics	CG = 14	HFrEF = 13	p
Age (years)	65 ± 5	64 ± 8	0.66
Height (cm)	167 ± 11	171 ± 12	0.28
Weight (kg)	72 ± 15	79.5 ± 9	0.45
Ejection fraction (%)	63.4 ± 6.23	32.7 ± 5.25	0.17
Women	0 (0)	2 (15.4)	0.78
**Ethnicity, n (%)**			
Black	3 (21.4)	3 (23)	1.00
White	11 (78.6)	10 (77)	0.87
**NYHA class, n (%)**			
I	-	1 (7.7)	-
II	-	9 (69.2)	-
III	-	3 (23)	-
**HF etiology, n (%)**			
Ischemic	-	4 (30.8)	-
Non-ischemic	-	9 (69.2)	-
Left ventricular ejection fraction (%)	-	32.7 ± 5.2	-
**Medications, n (%)**			
Warfarin	-	1 (7.7)	-
ACE inhibitors/BRA	-	13 (100)	-
Beta-blockers	-	13 (100)	-
Diuretic	-	7 (53.7)	-
Digoxin	-	7 (53.8)	-
Isosorbide	-	2 (15.4)	-
Antidiabetic	-	4 (30.7)	-
Aspirin	-	3 (23)	-
Simvastatin	-	4 (30.7)	-

Data are shown in mean and standard deviation or frequency (absolute and relative); HF: heart failure; NYHA: New York Heart Association; ACE: angiotensin-converting-enzyme; ARBs: angiotensin II receptor blockers.

### 6-minute walk test

#### Acceleration and deceleration – CO, HR, and SV

CO acceleration was significantly different between groups (HFrEF: 1.89 ± 1.39 l.min ^-1^ .s ^-1^ ; CG: 4.59 ± 2.75 l.min ^-1^ .s ^-1^ , p<0.01). In contrast, CO deceleration was not different between groups (HFrEF: 0.62 ± 1.39 l.min ^-1^ .s ^-1^ ; CG: 1.94 ± 2.11 l.min ^-1^ .s ^-1^ , p=0.07) ( Figure 3 ). Likewise, HR acceleration was significantly different between groups (HFrEF: 12 ± 12 bpm.s ^-1^ ; CG: 24 ± 15 bpm.s ^-1^ , p=0.039), whereas HR deceleration was not different between the groups (HFrEF: 9 ± 8 bpm.s ^-1^ ; CG 11 ± 9 bpm.s ^-1^ , p=0.385, respectively) ( Figure 4 ). In contrast, both VS acceleration and deceleration were not statistically different between groups (HFrEF: 15.51 ± 14.38 ml.s ^-1^ ; CG: 25.12 ± 15.65 ml.s ^-1^ , p=0,110 and HFrEF 3.29 ± 9.01 ml.s ^-1^ ; CG: 8.85 ± 16.98 ml.s ^-1^ , p=0.304).

**Figure 3:** – Comparison of cardiac output (CO) acceleration and deceleration responses to six-minute walk test between patients with heart failure with reduced ejection fraction (HFrEF) healthy individuals (control group).

**Figure 4:** – Comparison of heart rate (HR) acceleration and deceleration responses to six-minute walk test between patients with heart failure with reduced ejection fraction (HFrEF) healthy individuals (control group).

#### Conventional measures

Table 2 lists the outcomes traditionally measured during 6MWT. Compared to participants in the CG, patients with HFrEF walked a shorter distance, with a similar HR at rest and during the test, but showed a lower HR during the first minute of recovery. Patients with HFrEF showed significantly lower peaks for SV and CI values ( Table 3 ).

**Table 2:** – Traditional parameters measured during the six-minute walk test

	CG (n= 14)	HFrEF = 13	p

	Mean ± SD or Median (IQR)	Mean ± SD or Median (IQR)	
Walked distance (m)	559.50 ± 61.36	395.50 ± 87.63	< 0.01
HR baseline (bpm)	72 ± 8	71 ±9	0.927
HR maximum (bpm)	112 (108;138)	116 (91;146)	0.607
HR change (bpm)	43 (31;67)	48 (23;65)	0.837
1-minute HR recovery (bpm)	15 ± 9	8 ± 11	0.097

Bpm: beats per minute; HR: heart rate; CG: control group; HFrEF: heart failure with reduced ejection fraction group; IQR: interquartile range.

**Table 3:** – Hemodynamic parameters measured by signal-morphology impedance cardiography during six-minute walking test

	CG (n= 14)	HFrEF = 13	p

	Mean ± SD or Median (IQR)	Mean ± SD or Median (IQR)	
SV baseline (ml)	83.77 ± 16.48	51.28 ± 13.78	< 0.01
SV maximum (ml)	147.03 ± 28.16	100.13 ± 27.96	< 0.01
SV change (ml)	60.36 (47.41; 69.82)	41.76 (30.76; 67.90)	0.13
1-min SV recovery (mL)	18.49 ± 13.38	5.34 ± 7.55	< 0.01
CO baseline (l.min ^-1^ )	5.94 ±1.05	3.64 ± 1.03	< 0.01
CO maximum (l.min ^-1^ )	16.35 (15.20; 17.70)	10.30 (7.31; 14.83)	< 0.01
CO change (l.min ^-1^ )	10.15 (8.82; 12.20)	7.10 (3.64; 10.90)	0.05
CI baseline (l.min ^-1^ .m ^-2^ )	2.97 (2.70; 3.41)	1.99 (1.68; 2.19)	< 0.01
CI maximum (l.min ^-1^ .m ^-2^ )	8.67 ± 2.46	6.12 ± 1.90	< 0.01
CI change (l.min ^-1^ .m ^-2^ )	5.61 ± 2.54	4.09 ± 2.03	0.09
1-min CI recovery (l.min ^-1^ .m ^-2^ )	1.77 ± 0.92	0.58 ± 0.70	< 0.01

CI: cardiac index; CO: cardiac output; SV: stroke volume; CG: control group; HFrEF: heart failure with reduced ejection fraction group; IQR: interquartile range; min: minute.

#### 6MWT walked distance, hemodynamic variables and functional class

The higher was CO acceleration, the longer was the distance walked during 6MWT (r=0.49, p=0.01). Baseline CI (r=0.60, p<0.01), peak CI (r=0.67, p<0.01), ΔCI (r=0.63, p<0.01), CI during the first minute of recovery (r=0.68, p<0.01), as well as SV during the first minute of recovery (r=0.50, p<0.01) significantly correlated with the distance covered during 6MWT.

The distance walked during 6MWT correlated with HR in the first minute of recovery (r=0.68, p<0.01) and NYHA class (r=0.62, p<0.01). Moreover, baseline and peak SV significantly correlated with distance walked during 6MWT (r=0.51, p=0.01, r=0.60, p<0.01, respectively). Baseline CO, peak CO, and ΔCO also significantly correlated with distance walked during 6MWT (r=0.52, p<0.01, r=0.67, p<0.01, r=0.61, p<0.01, respectively).

## Contribution of variables to cardiac output change

Changes to HR explained 64.3% of the ΔCO in the control group and 70.3% in patients with HFrEF. The contribution to ΔSV totaled 57.4% in the control group and only 22.9% in individuals with HFrEF. According to linear regression β coefficients, for each altered HR unit, ΔCO changed by 1.121 units in the control group and by 0.92 unit in the HFrEF group. Regarding ΔSV, each altered HR unit, ΔSV changed by 1.162 units in the control group and to 0.91 unit in the HFrEF group.

## Discussion

In the present study, we compared cardiodynamicW responses to 6MWT between HFrEF patients and healthy individuals. Our main finding is that HFrEF showed blunted hemodynamic responses to 6MWT, especially CO and HR acceleration when compared to controls. Additionally, we found significant differences in the distance walked during the 6MWT between the groups, reaffirming the expected reduction in the functional capacity of individuals with HFrEF. Furthermore, during recovery, HR response was lower in the HFrEF group, suggesting an impairment of the parasympathetic autonomic nervous system, which actually occurs in these patients ( Central illustration ).

Autonomic imbalance, characterized by sympathetic predominance, is a classical feature of HFrEF, with clinically relevant consequences. These include disease progression, development or deterioration of exercise intolerance, ventricular remodeling and arrhythmias, and premature death. The mechanisms underlying these processes and their relative time courses still need to be described.

To our knowledge, several studies have evaluated the hemodynamic profile of different diseases during 6MWT. ^15 , 21 - 23^ One study ^22^ assessed CO (but not SV) acceleration and deceleration in pulmonary hypertension. These variables are important for HFrEF, as they represent an imbalance in autonomic responses to exertion and recovery. ^24 , 25^ We found a significant difference in CO acceleration (p<0.01) but not in deceleration (p=0.07) between groups, and a similar behavior in HR acceleration (p=0.039) and deceleration (p=0.385). Both CO and HR acceleration and deceleration were lower in the HFrEF group compared with controls; these patients show a chronotropic deficit due to the disease *per se* and because of the pharmacological effect of beta-blockers (all patients were receiving in beta-blocker therapy).

Since acceleration and deceleration may represent sympathetic and parasympathetic activation, respectively, HFrEF shows greater sympathetic activation and sympathovagal imbalance, which corroborates the findings of our study, showing lower CO and HR acceleration in the HFrEF group. ^26 , 27^ Animal models have shown that sympathoexcitation and abnormal cardiovascular reflex function contribute to activate the sympathetic nervous system in HFrEF. ^28^ In contrast, little is known about the role of parasympathetic nerve activity in HFrEF. Hu et al. ^26^ showed that HR deceleration is an independent predictor of acute myocardial infarction and sudden cardiac death in HFrEF, constituting a stronger predictor than left ventricular ejection fraction and conventional HR variability measurements. Hu et al., ^26^ only evaluated HR acceleration and deceleration responses. Therefore, our study is the first to assess CO, HR, and SV acceleration and deceleration in HFrEF. Although this study failed to demonstrate a significant difference in CO deceleration between the groups, we found a clear tendency (P=0.07) of loss in the responses in the HFrEF group.

Regarding HR regulation, ventricular impairment in HFrEF can trigger distinct compensatory mechanisms, which initially increases neurohormonal activation of the sympathetic nervous and renin-angiotensin-aldosterone systems. ^29^ However, prolonged exposure to sympathetic activation may downregulate β-adrenergic receptors in cardiomyocytes, contributing to inotropic responses which can impair HR recovery after exercise. ^30^ These data are corroborated by our results. ^2^

We found lower baseline and peak SV values in HFrEF patients, when compared to controls. Both resting and exercising behavior in the HFrEF group represents a loss in ventricular contraction, *i.e* ., a reduction in SV in each systole. ^31^ In healthy individuals, the Frank–Starling principle is a physiological mechanism which increases SV to compensate for the initial reduction of ventricular contraction. ^32^ On the other hand, individuals with HFrEF display failures in this mechanism. The attendant reduction in cardiovascular reserve impairs and reduces ventricular contractility, decreasing SV. ^33^ This phenomenon also corroborates the findings of this study.

In addition, we observed significant differences in baseline and peak CI and CO values between groups, which may be explained by HR and SV compensatory mechanisms. ^21^ We found that ventricular impairment reduced CI and CO in individuals with HFrEF, leading to mechanisms that initially increase these variables to maintain end-organ perfusion. ^34^ However, after prolonged exposure to them, the myocardium undergoes remodeling, reducing ventricular inotropic capacity and SV, consequently affecting both CI and CO. ^2^

When subjected to submaximal exercise, individuals with HFrEF show lower responses to both increased and decreased VO _2_ than healthy individuals. ^35^ These patients may also show mild pulmonary hypertension, which may explain CO and SV behavior. ^22 , 36^ CO behavior may thus be due to imbalanced autonomic responses affecting SV. ^37^

We tested whether hemodynamic parameters, assessed via SM-IGC, correlated with the distance walked during 6MWT, and found that the greater the CO acceleration, the greater the distance walked (r=0.49, p=0.01). Moreover, individuals with the fastest HR recovery after the test were those who walked the longest distances. Even though this was not the main aim of the study, we found that SM-ICG hemodynamic assessment during 6MWT can provide interesting results that directly reflect functional capacity. In addition to the 6MWT distance, we found that NYHA functional class had a good association with maximum CO, CO deceleration, and HR during the first minute of recovery after the test. ^38^ As expected, we found different 6MWT distances between groups (p<0.01), corroborating previous research. ^39^

Finally, linear regression showed the impaired contribution of SV (22.9%) to CO changes in patients with HFrEFm and normal values in healthy controls (57.4% of SV contribution to CO change). In fact, oxygen pulse, a SV surrogate, was lower in patients with HFrEF (compared to healthy controls), as per previous studies. SV impairment, as represented by low oxygen pulse, may reflect insufficient systemic oxygen delivery during exercising and/or reflect impaired oxygen utilization due to reduced mitochondrial function. ^40 , 41^

Some limitations of this study must be addressed. First, the small sample size may have limited the ability to detect significant differences. However, the main aim of this physiological study was to evaluate the hemodynamic behavior of individuals with HFrEF during 6MWT, compare it to that of healthy individuals, and determine the relative contribution of CO, SV, and HR variations during the phases of the walking test. Secondly, our study was not designed to perform correlation or association tests of SM-ICG parameters with walked distance, NYHA functional class, and ejection fraction.

Although 6MWT is safe, inexpensive, and easily used to assess functional capacity in patients with HFrEF, it has both clinical and research limitations ^20 , 39^ (e.g., it fails to provide direct hemodynamic behavior data). Therefore, new technologies would be important to add information to 6MWT findings and help in the treatment of this ominous syndrome. Moreover, technological advances have allowed the development of a portable device to measure, noninvasively and in real time, a wide range of hemodynamic parameters, such as CO, SV, HR, and CI.

## Conclusion

This is the first study to show CO, HR, and SV acceleration and deceleration hemodynamic responses via SM-ICG in patients with HFrEF during 6MWT. Individuals with HFrEF showed impaired CO and HR acceleration during submaximal exercises compared to healthy controls, which may represent an imbalance in their autonomic response to exertion. Further studies are needed to test whether changes in CO, HR, SV, and CI during 6MWT can provide information on disease prognosis.
